# Cortical Thickness and Clinical Findings in Prescholar Children With Autism Spectrum Disorder

**DOI:** 10.3389/fnins.2021.776860

**Published:** 2022-02-07

**Authors:** Simona Lucibello, Giovanna Bertè, Tommaso Verdolotti, Martina Lucignani, Antonio Napolitano, Rosa D’Abronzo, Maria G. Cicala, Elisa Pede, Daniela Chieffo, Paolo Mariotti, Cesare Colosimo, Eugenio Mercuri, Roberta Battini

**Affiliations:** ^1^Pediatric Neurology Unit, Fondazione Policlinico Universitario A. Gemelli IRCCS, Rome, Italy; ^2^Dipartimento di Diagnostica per Immagini, Istituto di Radiologia, Università Cattolica del Sacro Cuore, Rome, Italy; ^3^UOC Radiologia e Neuroradiologia, Fondazione Policlinico Universitario A. Gemelli IRCCS, Rome, Italy; ^4^Medical Physics Unit, Bambino Gesù Children’s Hospital, IRCCS, Rome, Italy; ^5^Centro Clinico Nemo, Fondazione Policlinico Universitario A. Gemelli IRCCS, Rome, Italy; ^6^Department of Clinical and Experimental Medicine, University of Pisa, Pisa, Italy; ^7^Department of Developmental Neuroscience, IRCCS Fondazione Stella Maris, Pisa, Italy

**Keywords:** autism spectrum disorder (ASD), cortical thickness, pre-scholar child, MRI, neuropsychological, local gyrification index

## Abstract

The term autism spectrum disorder (ASD) includes a wide variability of clinical presentation, and this clinical heterogeneity seems to reflect a still unclear multifactorial etiopathogenesis, encompassing different genetic risk factors and susceptibility to environmental factors. Several studies and many theories recognize as mechanisms of autism a disruption of brain development and maturation time course, suggesting the existence of common neurobiological substrates, such as defective synaptic structure and aberrant brain connectivity. Magnetic resonance imaging (MRI) plays an important role in both assessment of region-specific structural changes and quantification of specific alterations in gray or white matter, which could lead to the identification of an MRI biomarker. In this study, we performed measurement of cortical thickness in a selected well-known group of preschool ASD subjects with the aim of finding correlation between cortical metrics and clinical scores to understand the underlying mechanism of symptoms and to support early clinical diagnosis. Our results confirm that recent brain MRI techniques combined with clinical data can provide some useful information in defining the cerebral regions involved in ASD although large sample studies with homogeneous analytical and multisite approaches are needed.

## Introduction

According to the *Diagnostic and Statistical Manual of Mental Disorders* (DSM-5), autism spectrum disorder (ASD) is a neurodevelopmental disorder, characterized by the presence of impaired social communication and unusually repetitive behaviors or restricted interests (American Psychiatric Association, 2013).

However, the term ASD includes a wide variability of clinical presentation (Lord et al., 2000), which seems to reflect a multifactorial etiopathogenesis, still unclear, encompassing different genetic risk factors and susceptibility to environmental factors (Dietert et al., 2011; Tordjman et al., 2014).

Over the years, disruption of brain development and the maturation time course has been recognized as mechanism of autism (Amaral et al., 2008), suggesting the existence of common neurobiological substrates, such as defective synaptic structure and aberrant brain connectivity (Ecker et al., 2016).

Therefore, several studies have assessed the utility of brain magnetic resonance imaging (MRI) in the evaluation of region-specific structural changes and quantification of specific alterations in gray (GM) or white matter (WM) [in terms of cortical thickness, gyrification index, GM surface and volume (Pagnozzi et al., 2018)].

The identification of an MRI biomarker could provide insight into the underlying mechanisms of symptoms and could additionally provide crucial support in early clinical diagnosis and dividing patients, sharing common features, into different groups to tailor specific interventions.

In literature, consensus exists on increased growth of total GM and WM volume (Courchesne et al., 2001; Sparks et al., 2002; Hardan et al., 2006; Hazlett et al., 2011; Nordahl et al., 2012; Mahajan and Mostofsky, 2015; Ismail et al., 2016; Libero et al., 2016; Lucibello et al., 2019), which involves specific brain regions, such as temporal lobes (Lai et al., 2013; Ecker et al., 2014; Riddle et al., 2017; Pappaianni et al., 2018), frontal lobes (Ecker et al., 2014; Zhou et al., 2014; Eilam-Stock et al., 2016; Patriquin et al., 2016), or both (Katuwal et al., 2015; Postema et al., 2019).

A comparable number of studies finds opposite results, reporting decreased volumes of the frontotemporal (Hardan et al., 2006; Ecker et al., 2010; Mak-Fan et al., 2012), and parietal cortex (Ecker et al., 2010), significantly smaller right (Haznedar et al., 1997), and bilateral (Ecker et al., 2010; Jiao et al., 2010), anterior cingulate gyrus, and decreased cortical volume in the orbitofrontal cortex bilaterally (Ecker et al., 2012).

Recently, many studies have focused their efforts on measurement of cortical thickness (Doyle-Thomas et al., 2013; Khundrakpam et al., 2017; Pereira et al., 2018; Prigge et al., 2018), and among them, the one with the largest ASD population (Van Rooij et al., 2018), showed promising results. Specifically, the authors found complex developmental trajectories involving different brain regions with significant differences in terms of increased cortical thickness in the frontal cortex and decreased thickness in the temporal cortex between ASD patients and controls during adolescence. Other authors focus their study on cortical gyrification, suggesting the involvement of both genetic and non-genetic factors in definition of cortical gyral and sulcal patterns (Lohmann et al., 1999; White et al., 2002; Kates et al., 2009; Kremen et al., 2010; Mata et al., 2010; Docherty et al., 2015; Kuhn et al., 2016; Bernardoni et al., 2018; Duan et al., 2020; Kruggel and Solodkin, 2020). Although analysis of gyrification has led to the identification of different cortical regions involved in ASD, there is high heterogeneity across studies.

However, no studies have investigated the correlation between clinical, genetic, and radiological findings in a well-selected pediatric population.

### Aim of the Study

As part of an ongoing project collecting ASD preschooler data sets, we retrospectively selected MRI data sets and applied a semiautomatic brain segmentation methodology to investigate cortical thickness and gyrification. Correlations of cortical indexes and clinical features were subsequently performed.

## Materials and Methods

### Study Design and Clinical Assessment

A retrospective study was designed. The study group included 39 preschool-age children regularly followed at the Child Neurology Unit of the Gemelli Hospital (Rome, Italy) with a diagnosis of ASD. As part of our routine assessment since March 2016, patients referred to our unit with a suspect clinical diagnosis of ASD undergo a detailed clinical assessment, including a neurological examination, Leiter or Wechsler scales according to age and cooperation, a comprehensive neuropsychiatric assessment using parent-reported questionnaires’ (Child Behavior Checklist, CBCL), and autism-specific diagnostic tools, specifically Autism Diagnostic Interview Revised (ADI-R) and Autism Diagnostic Observation Schedule, second edition (ADOS2) (Lord et al., 1989, 1994). In addition, MRI is also routinely performed.

To have a relatively homogeneous cohort, the following exclusion criteria were used: (1) dysmorphic features with specific genetic syndrome identification, (2) presence of severe epilepsy, (3) presence of cerebral palsy or other major neurological signs, (4) malformations or other lesions at MRI.

The scores of all subscales of the ADI-R (social interaction, communication and language, restricted and repetitive behaviors) and ADOS2 (social affect score, restricted and repetitive behavior score, total score) were correlated with cerebral cortical thickness and cerebral cortical gyrification with the aim of finding a plausible neural substrate for the domains of deficit: impaired social communication and unusually repetitive behaviors or restricted interests.

### Study Design and Neuroradiological Protocol

All participants underwent MRI with a 1.5T Philips Ingenia Scanner (Philips Healthcare, Eindhoven, Netherlands). The sequence used for postprocessing was a T1-weighted 3-D-TFE in a sagittal orientation (TR = 9.8 ms, TE = 4.6 ms with a delay time of 650 ms after a 180° prepulse, flip angle = 10°, FOV = 200 mm × 222 mm, 1.0 mm slice thickness with no gaps, total of 150 slices per slab, matrix size = 200 × 222, NSA = 2 with an in-plane resolution of 1.0 × 1.0 mm^2^). This sequence is routinely acquired in our MRI protocol for children older than 2 years of age. ASD subjects were sedated with a general anesthesia with a halogenated agent while spontaneously breathing; contrast agent injection was never required. The written informed consent from a parent or guardian of children was obtained.

The quality of the structural MRI data was rated by an experienced neuroimaging researcher (TV) on a 3-point rating scale: 0 = no motion artifacts, excellent quality; 1 = few motion artifacts, fair quality; and 2 = moderate/severe motion artifacts, poor quality. Only data sets with scores of 0 were considered of adequate quality for research purposes. In case of imaging artifacts (ghosting, aliasing, chemical shift, and distortion), patients were excluded.

The raw 3-D T1 MRI data underwent automated processing for surface-based cortex reconstruction and volumetric segmentation using Freesurfer image analysis software (version 6.0.0), which is documented and freely available for download online^1^, installed on an OSX El Capitan 10.11.6.

The technical details of these procedures are described in prior publications; briefly, the processing pipeline includes motion correction and averaging (Reuter et al., 2010) of volumetric T1 weighted images, removal of non-brain tissue (Ségonne et al., 2004), automated Talairach transformation, segmentation of the subcortical WM and deep GM volumetric structure (including hippocampus, amygdala, caudate, putamen, ventricles) (Fischl et al., 2002, 2004) intensity normalization (Sled et al., 1998), tessellation of the GM/WM boundary, automated topology correction (Fischl et al., 2001; Ségonne et al., 2007), and surface deformation following intensity gradients to optimally place the GM/WM and GM/CSF borders (Dale and Sereno, 1993; Dale et al., 1999; Fischl and Dale, 2000). Cortical thickness was quantified as the closest distance from the GM/WM boundary to the GM/CSF boundary at each vertex. Cortical parcelation and thickness estimations were based on the Desikan–Killiany Atlas (Desikan et al., 2006), resulting in average cortical thickness in 34 cortical parcels per hemisphere. Procedures for the measurement of cortical thickness have been validated against histological analysis (Rosas et al., 2002) and manual measurements (Kuperberg et al., 2003; Salat et al., 2004).

Local gyrification index (LGI) was computed using the approach proposed by Lyu et al. (2018)^2^ that quantifies cortical gyrification within sulcal and gyral regions using a spatially varying kernel shape, able to adaptively encode cortical folding patterns. The proposed LGI is then computed within the adaptive kernel as a ratio of the cortical surface area and a fixed area on the outer hull (ρ = 316 mm^2^) (Lyu et al., 2018; Płonka et al., 2020).

The Ethics Committee of Policlinico Gemelli, Catholic University of Sacred Hearth, examined and approved the project study (February 2018).

### Statistical Analysis

We investigated differences in cortical parameter distributions (i.e., cortical thickness, CT, and local gyrification, LGI) among subjects grouped on the basis of ADOS2 and ADI-R domains scores. To this purpose, we first mapped vertex-wise CT and LGI values on a common spherical coordinate system using spherical transformation, and then we assessed differences among groups using permutation tests (1,000 permutations for all tests) based on the *t*-statistics, performed with the Permutation Analysis of Linear Models (PALM) FSL package (^3^ version 6.0). In particular, we used group age as a covariate to produce threshold-free cluster enhancement (TFCE) statistical maps, where the initial raw statistical images were enhanced using both the intensity of the data point and information from neighboring voxels (Mensen and Khatami, 2013). We detected group differences on both family-wise error (FWE) corrected and uncorrected *p*-value maps. Moreover, correlation analyses were evaluated vertex-wise between cortical parameters (CT and LGI) and several clinical variables, including social interaction (SI), communication (COM), and total scores (TOT, defined as SI + COM), together with their autism diagnostic interview (ADI) counterparts, i.e., ADI_SI, ADI_COM, repetition ADI (ADI_rep) and ADI_TOT as sum of the previously mentioned scores. Correlation analyses were performed testing Pearson correlation with PALM permutation test (1000 permutations).

## Results

After image quality check, five patients were excluded. Data from 34 patients, 4 females (11.8%) and 30 males (88.2%), were eventually included in the analysis. Their age range was between 3.4 and 6 years. All subjects of the sample selected had normal MRI findings and normal FMR1 and CGH array analysis.

Seven patient (20.6%) had a normal intellectual quotient, 12 (35.3%) a mild intellectual disability, and three (8.8%) a moderate intellectual disability.

About ADOS valuation, 5/34 ASD patients (14.7%) had a low level of severity, 20/34 (58.8%) had a medium level of severity, and 5/34 (14.7%) had a high level of severity (Table 1).

**TABLE 1:** Neuropsychological variables of the sample enrolled (34 patients).

	Mean	SD
Intellectual quotient	70.07	12.286
ADOS social affect score	10.81	3.894
ADOS repetitive behavior score	4.76	2.700
ADOS total score	15.41	4.790
ADI social interaction score	13.04	7.190
ADI communication score	8.08	3.049
ADI repetitive behavior score	6.08	3.006

### Correlations

#### Autism Diagnostic Interview – Social Interaction Domain

Statistical analysis showed a correlation between the subtest of ADI that investigates social interaction aims and thickness of different left-brain regions: fusiform (*p* = 0.046), lingual (0.049), posterior cingulate (0.048), pre-cuneus (*p* = 0.046), superior parietal (*p* = 0.033), inferior parietal (*p* = 0.033), superior temporal (*p* = 0.043), inferior temporal (*p* = 0.041), middle temporal (0.037), temporal pole (*p* = 0.041), and lateral occipital (0.041).

**FIGURE 1:**
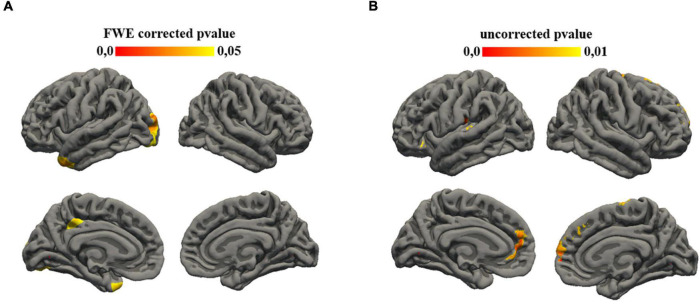
Statistical results mapped on the surface template. **(A)** Family-wise error (FWE) corrected *p*-value maps for cortical thickness ADI-social interaction correlation. Significance was set at 0.05. **(B)** Uncorrected *p*-value maps for gyrification ADI-social interaction correlation. Significance was set at 0.01.

A correlation was found also between the subtest of ADI that investigates social interaction aims and gyrification of different cortical regions: in left hemisphere insula (*p* = 0.01), rostral anterior cingulate (0.008), pars orbitalis (*p* = 0.009), superior frontal (*p* = 0.007), medial orbito-frontal (*p* = 0.006), lateral orbito-frontal (*p* = 0.008), precentral (*p* = 0.008), postcentral (*p* = 0.004), supramarginal (*p* = 0.007), transverse temporal (*p* = 0.009); in right hemisphere, superior frontal (*p* = 0.008), precentral (*p* = 0.007), paracentral (*p* = 0.009) (Figure 1).

#### Autism Diagnostic Interview – Communication Domain

**FIGURE 2:**
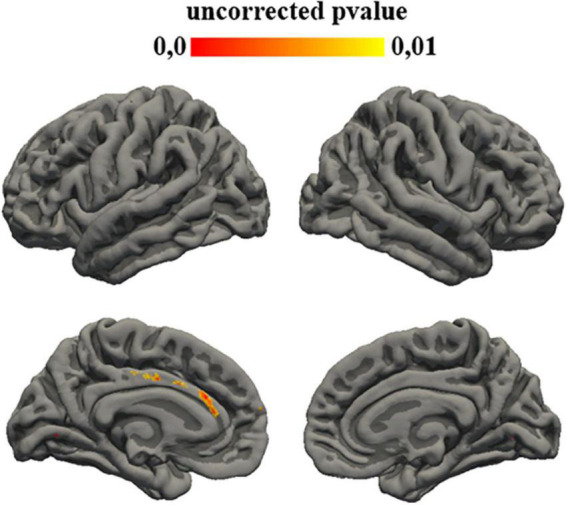
Uncorrected *p*-value maps for gyrification ADI-communication correlation. Significance was set at 0.01.

Statistical analysis showed a correlation between the subtest of ADI that investigates communication aims and gyrification of different left cortical regions: caudal anterior cingulate (*p* = 0.007), posterior cingulate (*p* = 0.008) (Figure 2).

#### Autism Diagnostic Interview – Repetitive Behavior Domain

**FIGURE 3:**
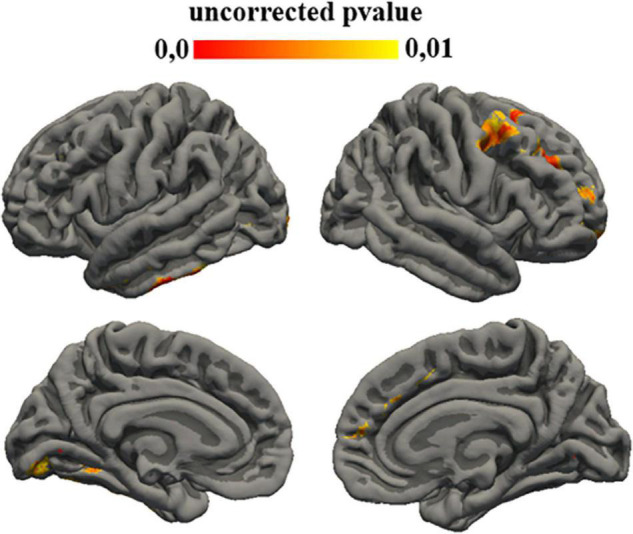
Uncorrected *p*-value maps for gyrification ADI-repetitive behavior correlation. Significance was set at 0.01.

Statistical analysis showed a correlation between the subtest of ADI that investigates social interaction aims and gyrification of different left regions: fusiform (*p* = 0.008), lingual (*p* = 0.008), para-hippocampal (*p* = 0.008), middle temporal (*p* = 0.006), inferior temporal (*p* = 0.005), lateral occipital (*p* = 0.008), but also with different right region: superior frontal (*p* = 0.006), frontal pole (*p* = 0.008), rostral middle frontal (*p* = 0.004), caudal middle frontal (*p* = 0.007), and precentral (*p* = 0.006) (Figure 3).

#### Autism Diagnostic Observation Schedule – Social Affect Score

Statistical analysis showed a correlation between the subtest of ADOS that investigates restricted and repetitive behavior aims and thickness of different left cortical regions: pre-cuneus (*p* = 0.007), posterior cingulate (*p* = 0.006), lateral orbito-frontal (*p* = 0.006), medial orbito-frontal (*p* = 0.005), precentral (*p* = 0.007), paracentral (*p* = 0.009), postcentral (*p* = 0.007), and superior parietal (*p* = 0.007).

A correlation was found also between the subtest of ADOS that investigates restricted and repetitive behavior aims and gyrification of left cortical regions: middle temporal (*p* = 0.008) and superior temporal (*p* = 0.008).

#### Autism Diagnostic Observation Schedule – Restricted and Repetitive Behaviors Score

**FIGURE 4:**
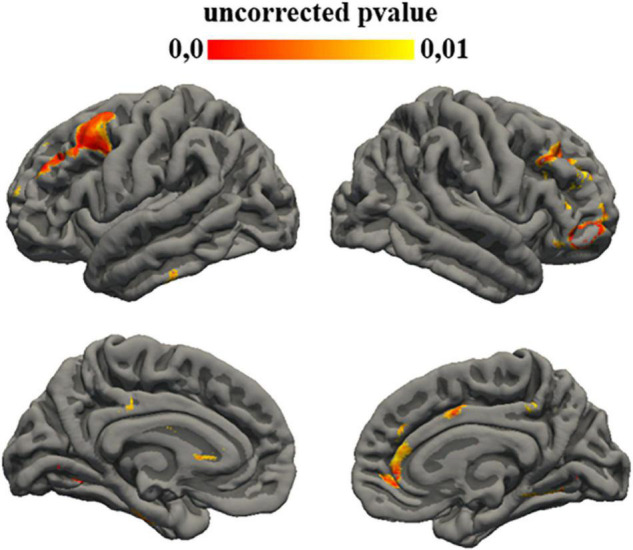
Uncorrected *p*-value maps for gyrification ADOS-restricted and repetitive behavior score correlation. Significance was set at 0.01.

Statistical analysis showed a correlation between the subtest of ADOS that investigates social affect aims and gyrification of different left cortical regions: fusiform (*p* = 0.008), lingual (*p* = 0.005), entorhinal (*p* = 0.008), para-hippocampal (*p* = 0.008), posterior cingulate (*p* = 0.009), superior frontal (*p* = 0.009), caudal middle frontal (*p* = 0.004), rostral middle frontal (*p* = 0.004), inferior temporal (*p* = 0.008) and also with different right regions: lingual (*p* = 0.008), para-hippocampal (*p* = 0.009), pars triangularis (*p* = 0.007), pre-cuneus (*p* = 0.009), posterior cingulate (*p* = 0.008), rostral anterior cingulate (*p* = 0.006), caudal anterior cingulate (*p* = 0.009), pars orbitalis (*p* = 0.004), superior frontal (*p* = 0.009), rostral middle frontal (*p* = 0.006), medial orbito-frontal (*p* = 0.006), and caudal middle frontal (*p* = 0.008) (Figure 4).

#### Autism Diagnostic Observation Schedule – Total Score

**FIGURE 5:**
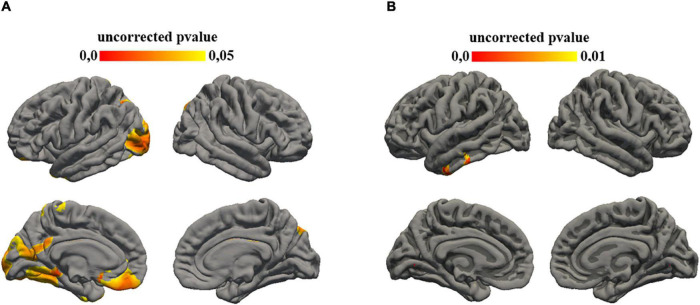
Statistical results mapped on the surface template. **(A)** Uncorrected *p*-value maps for cortical thickness-ADOS_total score correlation. Significance was set at 0.05. **(B)** Uncorrected *p*-value maps for gyrification-ADOS-total score correlation. Significance was set at 0.01.

Statistical analysis showed a correlation between the total score of ADOS and gyrification of left cortical regions: middle temporal (*p* = 0.009) and inferior temporal (*p* = 0.007) (Figure 5).

## Discussion

Autism is a complex and heterogeneous neurodevelopmental disorder with widely varied clinical features and a multitude of possible etiological factors.

Emerging evidence supports that ASD undergoes an atypical trajectory of brain maturation (Doyle-Thomas et al., 2013; Ecker et al., 2014) or abnormal lateralization of brain networks (Conti et al., 2015). Findings of atypical brain morphology in ASD, however, are highly heterogeneous and there are still not clear neuroanatomical markers to accurately identify individuals with ASD.

Whereas most neuroradiological studies on autism have focused on finding a specific pattern of brain involvement, only a few studies aimed to establish a correlation between clinical severity and cortical thickness (Doyle-Thomas et al., 2013). Additionally, many of these studies exclusively investigate well-known cortical areas involved in specific function, whereas we have tried to assess the correlation of several cortical areas to verify some correspondence with already known brain circuits. Whereas some results have reinforced well-known concepts already widely described in literature, others are quite difficult to frame.

The correlation between cingulate and frontal regions, particularly the left medial orbito-frontal area, with a social affect score of both ADOS and ADI-R scales is not surprising as the involvement of this area in social-affective processing is already described as part of the ventral social affective processing system (Belger et al., 2011). These results are consistent with what is already known about cortical gyrification morphology in neurodevelopmental disorders (Kohli et al., 2019).

Less clear is the correlation with other areas, such as the left lateral-occipital area, even if other authors have already shown this result in general in ASD patients (Kohli et al., 2019).

Among the results obtained, it is useful to underline a partial congruence between the results of the ADOS2 and ADI-R subtests investigating social affective behaviors and cortical gyrification index. This highlights how the two diagnostic tests support each other and how it is essential to always try to perform a complete patient assessment.

In the same way, it is easy to understand the correlation found between the restricted and repetitive behavior indices of both scales with gyrification of para-hippocampal, temporal, and middle frontal area.

Many human neuroimaging studies have indeed shown increased activation in ventral striatum and ventromedial frontal cortex in response to unexpected negative feedback that implies a change of behavior strategy (Glascher et al., 2009; D’Cruz et al., 2016; Sharda et al., 2017).

Conversely, it is unclear how the cingulate cortex could be involved in communication tasks although some neuroimaging studies suggest the association with this region and indirect language and social inferential capacity, such as the comprehension of non-literal language or construction-based pragmatic information (D’Cruz et al., 2011).

In general, the assessment of cortical areas based on the ADOS and ADI scores cannot be univocal. These scores, indeed, assess other skills, such as language or different non-verbal communication ability that could be underpinned by more cortical areas. Certainly, many of these areas, such as temporal and frontal or middle temporal areas, are already described as involved in patients with ASD.

This study confirms and expands previous results while reducing the variability of cohort patients as, in other studies a systematic selection of ASD subjects was not performed, and combining imaging with more detailed clinical data. The well-selected population of our study allowed us to reduce the variability observed between different ages.

Exclusion criteria, such as dysmorphic features or epilepsy or other major neurological signs, have led to less variability related to secondary factors, and focusing on only preschool-age patients has reduced the bias linked to the noted changes in the thickness of the cerebral cortex related to age (Shibata et al., 2010).

Neuroimaging studies indeed report numerous region-specific alterations in cortical thickness in patients with ASD. However, there are many inconsistent findings, and this is probably due to atypical CT developmental trajectories in ASD, characterized by decreased cortical thinning during early adolescence and increased thinning at later stages, involving mostly frontal and parietal areas (Mensen and Khatami, 2013; Ecker et al., 2014).

Limitations of our study include a small sample size, which reduces the power of statistical analysis.

Though the study has investigated the brain structure–function correlation with the aim of catching a possible clinical-related sensitivity marker at this age, the lack of controls constitutes a limitation of the study. The need for sedation during MRI scans, indeed, limits the opportunity to collect age-matched typical subjects.

Moreover, because pediatric brains are different in size and shape from adult brains commonly used as frameworks for spatial normalization (e.g., Talairach space), specific cortical areas could show correlation of deformation with age. In this context, several studies assessed the influence of age on various spatial normalization parameters (Wilke et al., 2002; Fonov et al., 2011), and consequently, our results should be taken with caution.

In conclusion, our results confirm that recent brain MRI techniques integrated to clinical data can provide some useful information in defining the cerebral regions involved in ASD although large sample studies characterized by homogeneous analytical and multisite approaches are needed.

## Data Availability Statement

The original contributions presented in the study are included in the article/supplementary material, further inquiries can be directed to the corresponding author/s.

## Ethics Statement

The studies involving human participants were reviewed and approved by Ethics Committee of Fondazione Policlinico Gemelli Hospital – Catholic University, Rome, Italy. Written informed consent to participate in this study was provided by the participants’ legal guardian/next of kin.

## Author Contributions

RB, SL, and EM contributed to conception and design of the study. SL, TV, and GB organized the database. EP, MC, DC, and RD’A contributed to investigation. AN and ML performed the statistical analysis. SL wrote the first draft of the manuscript. TV and AN wrote sections of the manuscript. PM, CC, and EM contributed to the supervision of manuscript. All authors contributed to manuscript revision, read, and approved the submitted version.

## Conflict of Interest

The authors declare that the research was conducted in the absence of any commercial or financial relationships that could be construed as a potential conflict of interest.

## Publisher’s Note

All claims expressed in this article are solely those of the authors and do not necessarily represent those of their affiliated organizations, or those of the publisher, the editors and the reviewers. Any product that may be evaluated in this article, or claim that may be made by its manufacturer, is not guaranteed or endorsed by the publisher.

## Footnotes

1
http://surfer.nmr.mgh.harvard.edu/

2
https://github.com/ilwoolyu/LocalGyrificationIndex

3
https://fsl.fmrib.ox.ac.uk/fsl/fslwiki
